# Lipid-Based Nanocarriers for The Treatment of Glioblastoma

**DOI:** 10.1002/anbr.202000054

**Published:** 2020-11-26

**Authors:** Nerea Iturrioz-Rodríguez, Rosalia Bertorelli, Gianni Ciofani

**Affiliations:** Smart Bio-Interfaces Istituto Italiano di Tecnologia Viale Rinaldo Piaggio 34, Pontedera 56025, Italy; Translational Pharmacology Istituto Italiano di Tecnologia Via Morego 30, Genova 16163, Italy; Smart Bio-Interfaces Istituto Italiano di Tecnologia Viale Rinaldo Piaggio 34, Pontedera 56025, Italy

**Keywords:** blood–brain barrier, glioblastoma multiforme, in vivo studies, lipid-based nanocarriers

## Abstract

Glioblastoma multiforme (GBM) is the most common and malignant neoplasia having origin in the brain. The current treatments involve surgery, radiotherapy, and chemotherapy, being complete surgical resection the best option for the patient survival chances. However, in those cases where a complete removal is not possible, radiation and chemotherapy are applied. Herein, the main challenges of chemotherapy, and how they can be overcome with the help of nanomedicine, are approached. Natural pathways to cross the blood–brain barrier (BBB) are detailed, and different in vivo studies where these pathways are mimicked functionalizing the nanomaterial surface are shown. Later, lipid-based nanocarriers, such as liposomes, solid lipid nanoparticles, and nanostructured lipid carriers, are presented. To finish, recent studies that have used lipid-based nanosystems carrying not only therapeutic agents, yet also magnetic nanoparticles, are described. Although the advantages of using these types of nanosystems are explained, including their biocompatibility, the possibility of modifying their surface to enhance the cell targeting, and their intrinsic ability of BBB crossing, it is important to mention that research in this field is still at its early stage, and extensive preclinical and clinical investigations are mandatory in the close future.

## 1 Introduction: Glioblastoma Multiforme

Glioblastoma multiforme (GBM) is the most common and malignant neoplasia stemming from the glia, with an annual incidence of around 1/33 330 and an estimated prevalence of 1-9/100 000 in most European and North American countries, with a higher incidence in men compared with women.^[1,2]^

Few studies have shown that Black people are less prone compared with other ethnic groups including Asians, Latinos, and Whites.^[2,3]^ Although the incidence rate of the disease is quite low, the median survival of patients with GBM is 12–15 months,^[4]^ resulting in a highly mortal disease. It can occur at any age, but 70% of the cases are reported in patients between 45 and 70 years of age. It is a rapidly growing tumor, composed of a heterogeneous mixture of poorly differentiated astrocytic cancer cells with frequent mitoses. Histologically, it is characterized by pleomorphism, necrosis, and vascular proliferation with an increase in the blood vessel diameter.^[5]^ GBM is a highly heterogeneous and very infiltrative disease, surrounded by edema and inflammation, with indistinct tumor margins, which make the complete surgical removal complicated.^[6]^

The first-line treatment is usually surgery, either to confirm the diagnosis with a biopsy or to remove as much as possible the tumor. Complete resection is rarely feasible, as tumor cells usually infiltrate the surrounding brain. Apart from surgery, radiotherapy and chemotherapy (nitrosoureas and temozolomide, TMZ) are usually performed. Radiation can be done as a postoperative therapy, with clear survival advantages, or as a prime treatment for unresectable tumors.^[4]^ It has been shown that patients treated with radiotherapy and chemotherapy have a longer overall survival compared with those treated with radiotherapy alone.^[7]^ Although the standard chemotherapy treatment scheme includes temozolomide, in the last years, different treatments have been studied. As GBM is one of the most vascularized tumors, antiangiogenic therapies have been developed, such us, among others, gene therapy blocking the vascular endothelial growth factor (VEGF)-dependent pathway,^[8]^ or bevacizumab, a humanized IgG1 monoclonal antibody that selectively binds and blocks VEGF.^[9]^ Besides the aforementioned therapies, gene therapy, immunotherapy,^[10]^ vaccine therapy,^[11]^ or stem cell therapy^[12]^ are also being studied.

## 2 Main Challenges in Chemotherapy

**Figure 1:**
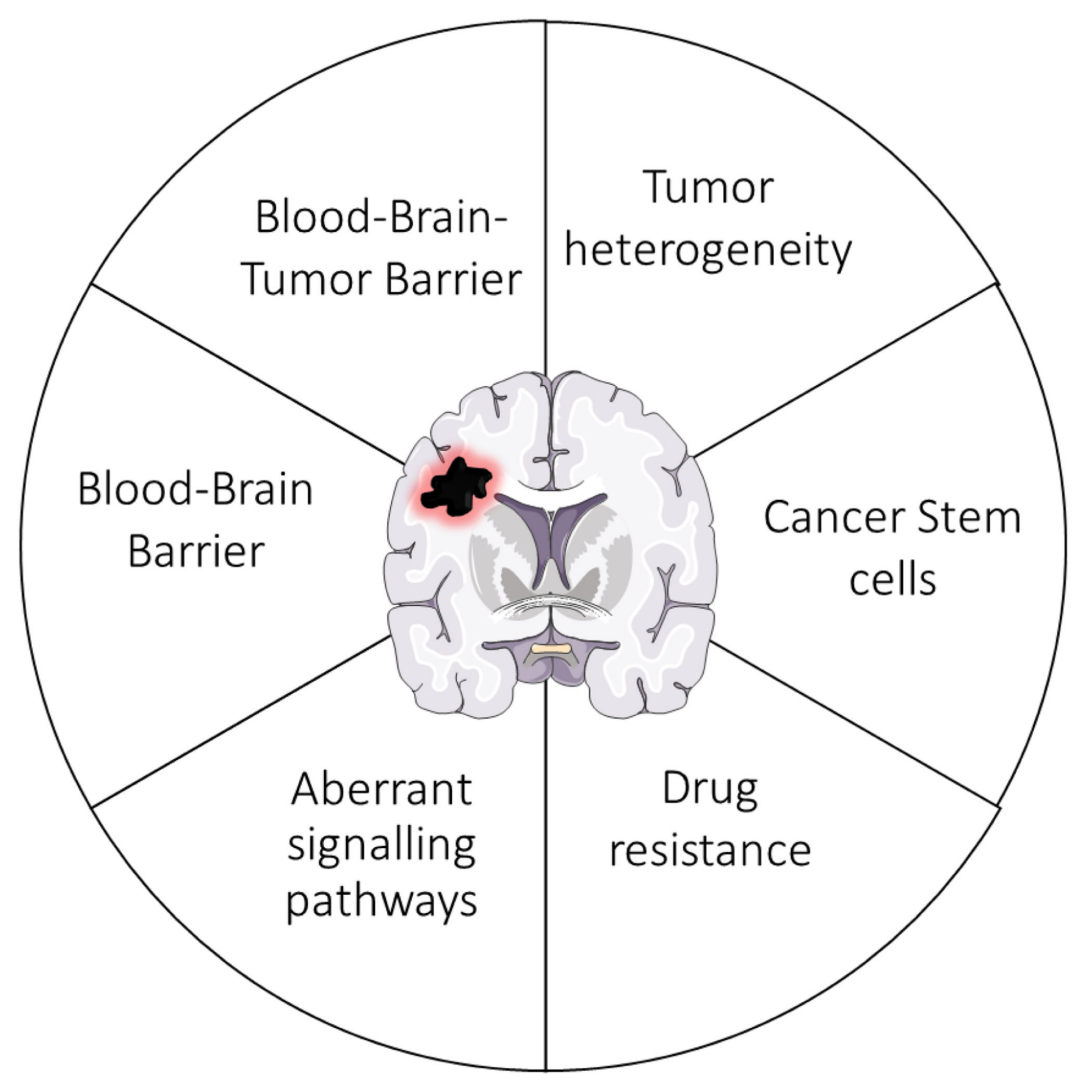
Representation of the main challenges in the chemotherapeutic treatment of glioblastoma.

Despite several strategies for GBM treatment, common challenges to overcome drug delivery include the presence of cancer stem cells,^[13]^ the high heterogeneity of the tumor,^[14]^ drug resistance,^[4]^ the aberrant signaling pathways,^[15]^ and most importantly, the existence of physical barriers, the blood–brain barrier (BBB) and the blood–brain–tumor barrier (BBTB)^[4,16]^ (Figure 1).

### 2.1 Cancer Stem Cells and Tumor Heterogeneity

Cancer stem cells initiate the tumor and drive its progression forward. In the advanced phases of GBM, hyperproliferation combined with increased genetic instability occurs in the cells, distinguishing clonal subpopulations.^[17]^ Even a single tumor mass has an intrinsic mosaicism: tumor clones emerge, drift, and branch following evolutionary dynamics, that are even fostered by generalized therapies.^[14]^ Secondary mutations generate more genetic variation among proliferative cells, the frequency of which changes not only randomly, yet it is also driven by environmental forces, such as therapy. For instance, an antitumoral drug might preferentially kill a specific cell population, allowing the expansion of non-drug-sensitive cells. Thus, it is particularly important to develop strategies that are toxic for all tumor cells and not for the healthy ones.

### 2.2 Aberrant Signaling Pathways and Drug Resistance

The development of malignant glioblastoma is due to the progressive accumulation of multiple intracellular events. These major events are 1) loss of cell cycle control, 2) overexpression of growth factors, 3) angiogenesis, 4) invasion and migration to surrounding tissues, 5) abnormalities in apoptosis, and, last but not least, 6) genetic instability.

Normal cells have a strict control, with different cell cycle checkpoints that ensure their correct proliferation. However, in malignant gliomas, these checkpoints are modified, with alterations of at least one component of the G1-S phase transition checkpoint.^[18]^

The overexpression of growth factors and their receptors is another important event that allows the development of malignant cancers. Among these factors we can include the epidermal growth factor receptor (EGFR), the platelet-derived growth factor (PDGF), the basic fibroblast growth factor (FGF), the transforming growth factor (TGF)-α, and the insulin-like growth factor (IGF)-1. In GBM specifically, EGFR and PDGF have been well characterized.^[19,20]^

Angiogenesis is another important characteristic of malignant GBM. As mentioned before, GBM is a vascular tumor, with a high rate of microvascular growth. Among the angiogenic molecules that can be found overexpressed in GBM, VEGF is one of the most common.^[18]^

An additional important feature of GBM is cell invasion and migration through infiltration of the surrounding tissues. To make this possible, cells have to express several extracellular matrix molecules and cell surface receptors,^[21]^ that include molecules that mediate interactions between the microenvironment and cytoskeleton, such as adhesion molecules and proteases.^[22]^

Glioma cells may develop malignancy not only changing their proliferation pathways, but also avoiding apoptosis. Different genes are involved in the apoptosis, but among them, p53 is the most known one. In normal conditions, when the damage in DNA is irreparable, p53 can activate the expression of proapoptotic genes, triggering apoptosis, but in tumoral cells this gene is deregulated.^[23]^ p53 can not only regulate the apoptosis, yet also plays a key role in maintaining the genomic stability. Thus, alterations in this gene can trigger further genomic damage, allowing the selection of more malignant clones.^[24]^

The intratumor heterogeneity may be responsible not only for the malignancy of GBM, but also for the drug resistance. Tumorinitiating cells can be responsible for the resistance to chemotherapy and even to radiotherapy, altering their mechanisms, such as, antiapoptotic signaling pathways, DNA damage response pathways, and overexpression of drug efflux transporters, among others.^[4,25,26]^ Moreover, the infiltrative nature of cells may also result into tumor resistance to radiation-combined therapies.^[27]^ For instance, the Wnt pathway is preferentially activated in postradiated cells. In the absence of Wnt signals, β-catenin forms a complex with E-cadherin participating in the cell– cell adherence junction formation. During tumor formation, Wnt/β-catenin signaling is activated, assuring the enlargement of the tumoral mass and eventually the spread of metastases.^[28,29]^ Another example of aberrant signaling pathway is PI3K/Akt, which is commonly hyperactivated in GBM.^[30]^ This activation can be achieved by multiple mechanisms, including activation of upstream growth factors, mutations of PI3K, overexpression of Akt, inactivation of tumor suppressors such as the phosphatase and tensin homolog (PTEN), or by the Wnt pathway.^[27]^ It has been shown that the hyperactivation of the pathway triggers cell survival, proliferation, invasion, and angiogenesis of GBM.^[30–32]^ The Hedgehog signaling pathway seems to be very important in the regulation of the stemness, selfrenewal, growth, and survival of GBM stem cells.^[33]^ In addition, the overexpression of TGF-β has an effect on the glioma microenvironment, affecting extracellular matrix deposition, angiogenesis, and invasion.^[34]^

### 2.3 The Blood–Brain Barrier

**Figure 2:**
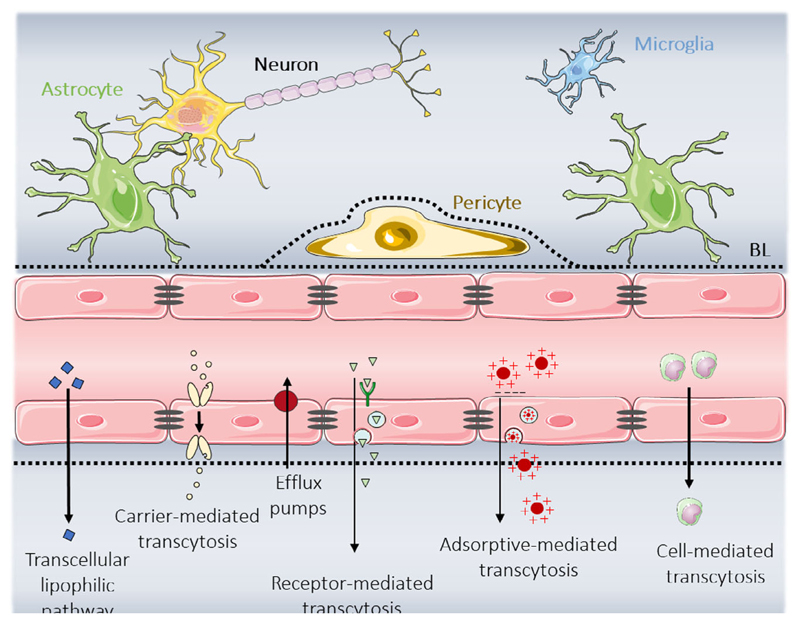
Scheme representing the BBB components: astrocytes, pericytes, endothelial cells connected by tight junctions (in grey), and the basal lamina (BL in black). At the bottom of the figure, the main transport mechanisms to cross the BBB are represented.

The BBB plays a critical role in brain homeostasis, protecting it from toxic substances and enabling the passage of nutrients. It is composed of endothelial cells that are closely sealed by tight junctions. These cells are surrounded by astrocytes and pericytes through the basal lamina (**Figure 2**, top).

The tight junctions in the endothelial cells limit the paracellular diffusion of water-soluble agents. Moreover, the lipids of the cell membrane and the presence of transport systems in the abluminal (brain side) and luminal (blood side) compartment enable the passage of different substances through different transport mechanisms (Figure 2, bottom). Small lipophilic molecules, O_2_, CO_2_, nicotine, steroid hormones, or alcohol can cross the BBB through the transcellular lipophilic pathway.^[35]^ Other molecules such as nutrients and ions (glucose, vitamins, electrolytes, amino acids, or nucleosides, among others) pass through the carrier-mediated transcytosis (CMT).^[36]^ The receptormediated transcytosis (RMT), which involves specific receptors, serves to transport low-density lipoprotein (LDL),^[37]^ transferrin (Tf),^[38]^ and insulin.^[39]^ In addition, other molecules such as cationic proteins or cell-penetrating peptides (CPPs) can cross the BBB by adsorptive-mediated transcytosis (AMT),^[40]^ due to electrostatic interactions with anionic sites of the endothelial cell membranes. The last type of transport is the cell-mediated transport. Stem cells and immune cells, such as macrophages and monocytes, can cross the BBB.^[41–43]^ However, even if a substance enters the brain, it can be extruded back to the blood circulation by efflux pumps. These are natural protective mechanisms of the brain to avoid exposure to foreign molecules. The ATP-binding cassette transporters, such as the multidrug resistance protein or the *p*-glycoprotein, are the most known transport carriers.^[35,44]^

In pathological conditions, the morphology and the physiology of the BBB are altered. Nevertheless, this does not imply the loss of efflux pumps. GBM patients present a disrupted, variable, and heterogeneous BBB, with intact regions that may be enough to limit the access of drugs to the tumor cells.^[4]^

### 2.4 The Blood–Brain–Tumor Barrier

The intensity of the malignancy of GBM alters the structure, the function, and the organization of the BBB. The BBTB is formed following the disruption of the tumor basal lamina and tumor deterioration. The transformation from a low-grade tumor to a more malignant one triggers the invasion of the nearby healthy brain tissue and the damage of the BBB, being replaced by the BBTB. Moreover, this tumor growth increases the amount of O_2_ and nutrients required, inducing the overexpression of VEGF and the angiogenesis in hypoxic areas.^[4]^ GBM is known to be one of most vascularized tumors, due to the growth of abnormal lymphatic vasculature presenting abnormal endothelial hyperplasia, pinocytic vesicles, fenestration, and opening or loss of tight junctions between endothelial cells.^[4,45]^ While these changes increase the permeability of the BBTB, the specificity of the glioma and the cranial microenvironment makes malignant gliomas less permeable.^[26,46]^

## 3 Nanomedicine to Overcome the GBM Therapeutic Challenges

Nowadays, chemotherapy is insufficient to treat GBM. Thus, it urges developing new systems that will overcome the challenges mentioned in the previous section. Nanomedicine has gained attention in the past two decades due to different advantages in the application of chemotherapeutic drugs to GBM. The encapsulation of drugs improves their solubility and stability and minimizes their side effects. Most of the chemotherapy drugs are hydrophobic molecules, making difficult their systemic administration.^[47]^ Thus, their encapsulation in a nanomaterial would ensure better transport and controlled release to the targeted cells or tissues^[48]^ and would help to cross the BBB and the BBTB and reach GBM by passive or active targeting processes.^[35,49]^

### 3.1 Passive Targeting to GBM

The enhanced permeability and retention (EPR) effect is the mechanism by which small molecules or nanoparticles accumulate in tissues that offer an increased vascular permeability and impaired lymphatic drainage, as it occurs with inflammation or cancer.^[50]^ Although preclinical studies suggest a slight accumulative effect in GBM,^[51]^ it has not been proved in patients.^[4]^ As passive targeting seems to be not so efficient, different active targeting strategies have been developed.

### 3.2 Active Targeting to Cross the BBB

**Figure 3:**
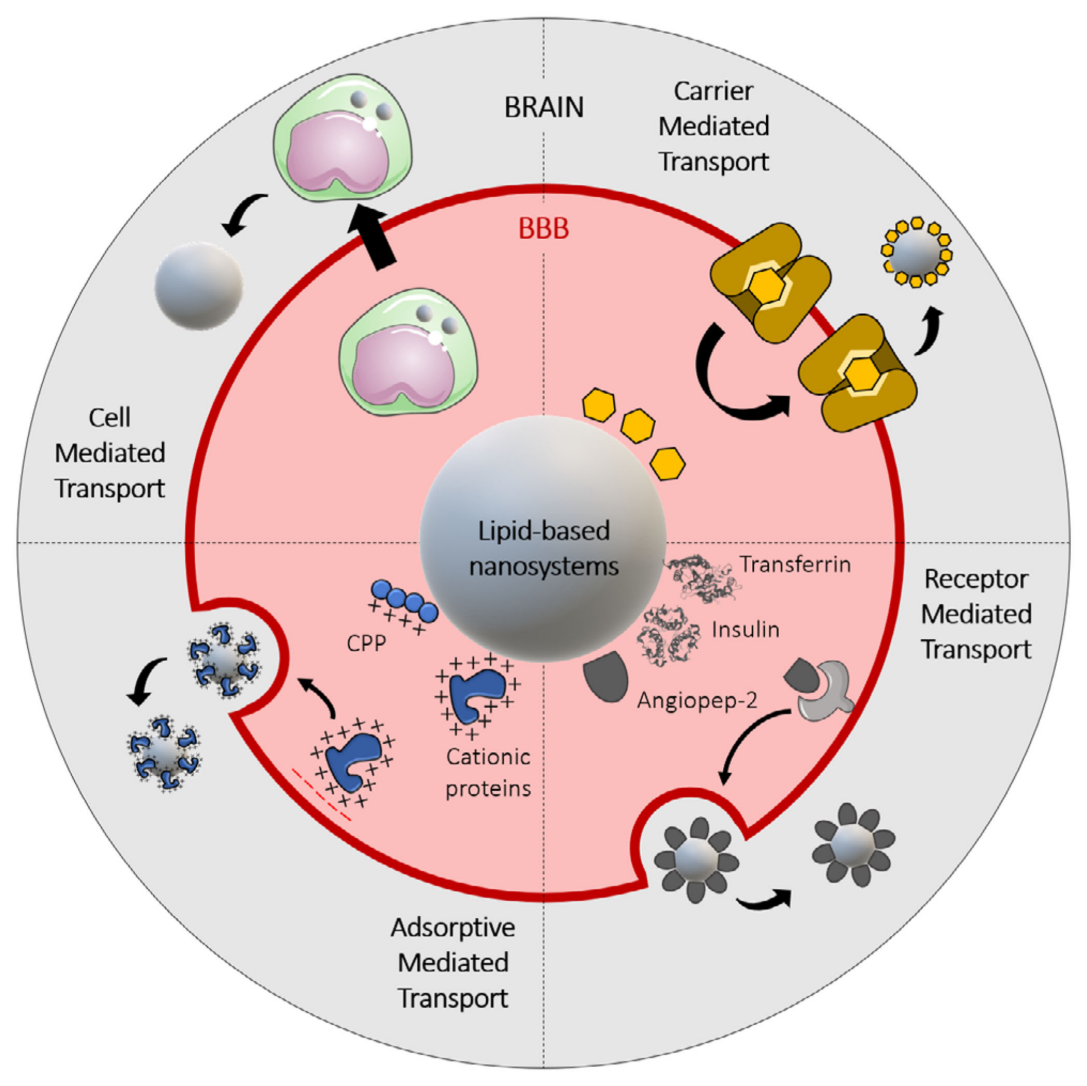
Schematic representation of the possible surface functionalization of lipid-based nanosystems to achieve an active targeting to cross the BBB or the BTBB.

The main objective of active targeting, achieved by modifying the surface of the nanomaterial, is to increase the lifetime of the system in the blood circulation and to improve the cell uptake and the therapeutic effect of the drug, while decreasing its systemic toxicity. In the case of GBM, the first obstacle to overcome will be the BBB: to cross the BBB, the nanocarrier surface can be modified (**Figure 3**) with ligands that will interfere with the endogenous transport mechanisms^[4,52]^ (Section 2.3, Figure 2).

#### 3.2.1 Carrier-Mediated Transcytosis

This pathway transports nutrients and hormones such as glucose and glutathione through the BBB. The most common transporter is the glucose transporter 1 (GLUT1) that is overexpressed in the endothelial cells of the BBB. Different systems have been developed targeting the GLUT1 with glucose-integrated liposomal formulations,^[53,54]^ but none of them achieved a high accumulation. Other strategies envisioned to target GLUT1 through mannose and its analogous, with a higher affinity for the transporter.^[55]^ An example of this formulation has been provided by Singh and collaborators,^[56]^ that modified the surface of solid lipid nanoparticles (SLNs) with a mannose-derived ligand, *p*-aminophenyl-α-D-mannopyranoside (MAM). Moreover, they encapsulated docetaxel and studied the pharmacokinetics and biodistribution, demonstrating an increment in the concentration of drug in the brain compared with the free drug. Ying et al.^[57]^ studied the efficacy of MAM in liposomes loaded with daunorubicin, again demonstrating an increase in the amount of drug in the brain, thus enhancing the passage through the BBB. In a more recent study conducted by Wang et al.,^[58]^ curcumin and quinacrine were encapsulated in liposomes functionalized with MAM; they demonstrated that mice treated with this formulation have a higher survival rate due to GBM growth inhibition.

#### 3.2.2 Receptor-Mediated Transcytosis

This type of mechanism allows for the transport of bigger molecules through specific receptors of the luminal side of the BBB, such as LDL receptors, Tf receptor 1 (TfR1), and scavenger receptors class B type 1. In the study conducted by Muntoni et al.,^[59]^ SLNs with methotrexate were functionalized with Tf or insulin. They demonstrated in Wistar rats a higher accumulation in the brain when they used both targeting proteins.

In addition to the mentioned ligands, different peptides are being used such as fragments of ApoB, ApoE, and angiopep-2. For instance, angiopep-2 might be a promising targeting moiety. Pucci and collaborators^[60]^ observed how the targeting efficiency of lipid-based magnetic nanovectors was increased when conjugated with angiopep-2 and how the transport through a BBB in vitro model was improved. The study conducted by Kadari et al.^[61]^ presented solid lipid particles loaded with docetaxel and functionalized with angiopep-2 as well. They showed that using their system the drug was accumulated at higher doses in mice brains if compared with the administration of the free drug. In addition, the survival rate of the mice was increased from 24 to 39 days, showing not only a better targeting, yet also a higher efficacy of the drug.

Another interesting strategy to overcome the BBB is the use of peptide shuttles. These systems have different advantages, including their low risk of immunogenicity, their simple synthesis, and the opportunity to introduce non-natural amino acids. The latters are designed to be protease-resistant peptides, so that their half-life time in blood circulation increases.^[62–66]^ A recent study conducted by Bukchin and collaborators^[67]^ demonstrated how peptide-modified nanoparticles not only present an increased accumulation of the nanoparticles in the brain, but also a higher permeability rate.

#### 3.2.3 Adsorptive-Mediated Transcytosis

This type of transport is based on the electrostatic interactions between positively charged substrates and the negative charge of the cell membrane. This mechanism is non-specific and many peptides and proteins can be transported,^[68]^ such as CPP and cationic proteins, for instance.^[35]^ However, using cationic proteins not only increases the uptake at brain level, but also in the kidney and in the liver, thus increasing the plasma clearance.^[69]^

Several studies have combined different transport strategies such as RMT and AMT, functionalizing the vector with Tf and with a CPP. Lakkadwala et al.^[70]^ functionalized liposomes loaded with doxorubicin (DOX) and erlotinib with transferrin and two CPPs (TAT and QLPVM). They demonstrated an accumulation in the brain 10 and 2.7 times higher for doxorubicin and erlotinib, respectively, compared with free drugs. The same research group^[71]^ studied similar liposomes functionalized with another CPP, and not only showed a better brain accumulation of the drugs in nude mice, but they also could increase the survival rate of mice and show a decrease in the tumor size.

#### 3.2.4 Cell-Mediated Transcytosis

Although previous pathways (CMT, RMT, and AMT) are promising strategies, none of them are brain exclusive; thus, a more specific transport has gained much attention: the cell-mediated transcytosis. Natural stem cells, mesenchymal stem cells, macrophages, and exosomes seem to have an intrinsic tumor-homing capacity.^[43,72,73]^ For instance, the study performed by Xue et al.^[74]^ demonstrated that neutrophils carrying liposomes with paclitaxel could penetrate brain and suppress glioma recurrence in cases where the mouse tumor was removed by surgery. The inflammatory factors released after the surgery guide neutrophils to the inflamed brain, and at the same time, these inflammatory signals make neutrophils release the liposomes. This study showed that the growth of recurrent tumors was slowed down, improving the survival rate of the mice; however, this system did not inhibit the regrowth of tumors.

### 3.3 Active Targeting to the GBM

In active targeting, nanovector surfaces are modified with ligands that interact with overexpressed receptors in GBM. Different structures can bond to the nanocarriers, including oligonucleotides or peptides.^[75]^ The ligands can be targeted to different cells such as glioma stem cells or glioblastoma cells and also to the extracellular matrix.^[76]^

In the case of extracellular matrix, its degradation is mediated by metalloproteases (MMP), secreted by tumor and stromal cells. MMP1 is the enzyme that initiates the breakdown of collagen, triggering the infiltration of glioma cells to the normal tissue. MMP1 is the main targeting moiety of nanovectors.^[15]^ The first study targeting MMP1 was conducted by Hatakeyama et al.^[77]^ They used an antibody against MMP1 attached to DOX-loaded liposomes and demonstrated an improvement in the cell uptake of the nanocarriers in vitro. Tenascin-C is another glycoprotein found at high levels in the extracellular matrix during fetal development, wound healing, atherosclerosis, psoriasis, and tumor growth.^[78,79]^ The aptamer TTA1 has been proved in GBMbearing nude mice, showing a rapid clearance from the blood and a good uptake into the tumors.^[80]^ Although this aptamer has not been attached to lipid-based nanoparticles, it might be a good targeting strategy.

The most common approach in targeting GBM is addressing overexpressed receptors in glioma cells. For instance, interleukin 13 (IL-13) peptide can specifically bind with high affinity to IL-13 Rα2, a tumor-specific receptor overexpressed in GBM.^[81]^ A study^[82]^ showed that nanoparticles modified with IL-13 were accumulated at higher levels in the tumor, because of an increased internalization of the nanoparticles in glioma cells. Another common example of the overexpressed receptor is EGFR, which is highly expressed in more than 40% of GBM cases.^[83]^ The study conducted by Høg Mortensen et al.^[84]^ used liposomes conjugated with an anti-EGFR antibody. They tested these nanocarriers in vivo and showed that α-hEGFR-liposomes were accumulated at a higher level in glioma cancer cells with respect to control liposomes.

### 3.4 Dual Targeting

Although single targeting systems seem to be promising, they often show inadequate efficiency and specificity to brain tumor. Thus, dual targeting to both BBB and glioma cells could enhance the nanovector accumulation. This can be achieved using a ligand that interact with both BBB and glioma cells, such as the LDL receptor-related protein (LRP), Tf, or the α7 nicotinic acetylcholine receptor.^[85–87]^ In the study conducted by Zheng et al.,^[87]^ a peptide that binds to α7 nicotinic acetylcholine receptors has been used, attached to the liposomal surface. This peptide is overexpressed not only by the endothelial cells of the BBTB, yet also in glioma cells and in tumor-associated macrophages. Using this targeting molecule, they achieved a “threebirds-one-stone” delivery strategy, transporting the system to endothelial cells, glioma cells, and to macrophages.

Another strategy for dual targeting is to use different ligands that will separately target BBB and GBM.^[57,88]^ A recent study led by Seok et al.^[88]^ exploited liposomes with two targeting moieties attached to their surface: on one hand, angiopep-2 was used to achieve both BBB transcytosis and GBM targeting and, on the other hand, they used an anti-CD33 monoclonal antibody to reach stem cells. They studied the system in vivo, demonstrating good targeting in U87MG tumor-bearing mice.

### 3.5 Glioma Preclinical Models

In cancer research, and thus also in GBM studies, it is crucial to use preclinical models, due to the fact that in vitro models lack the appropriate microenvironment and the heterogeneity characterizing cancer, and in general they are too simple to understand the pharmaceutical response of the treatment. In vivo models should be reproducible and stable in time, able to accurately predict novel therapeutic strategies in human GBM, weakly or non-immunogenic, and have similar histopathological features that closely resemble the human GBM.^[4]^ Among in vivo models we can find 1) chemically induced glioma models, where DNA alkylating agents are administrated to animals by intravenous, local, or oral administration; 2) xenograft transplantation models, where human GBM cells are transplanted in immunosuppressed or immunodeficient animals; and 3) genetically engineered mouse models, where the tumor is developed mutating driver genes.

Depending on the location of the cell transplantation, xenograft model can be distinguished in orthotopic models, if they are implanted in the nervous system, or ectopic models, if cells are transplanted into a different site than the origin of the cultured cells (usually hind legs). Within all the in vivo models, the most used one is the orthotopic model, due to its more realistic microenvironment.

## 4 Lipid-Based Nanovectors

**Figure 4:**
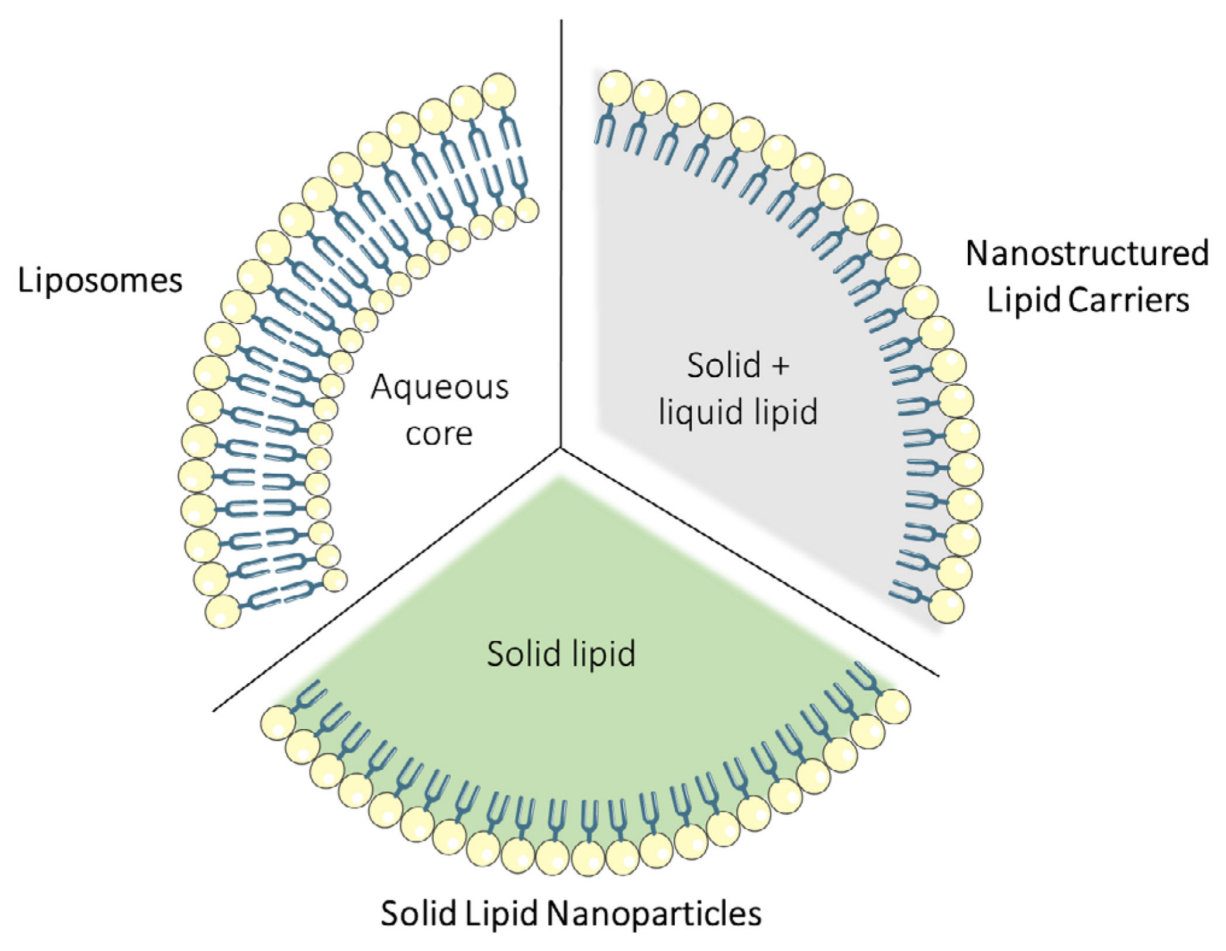
Schematic representation of the three main lipid-based nanosystems approached in this Review: liposomes, SLNs, and NLCs.

There are different nanomaterials that can be used to prepare drug delivery systems; thus, we can have, just mentioning some examples, carbon-based,^[89–91]^ silica-based,^[92,93]^ or lipid-based nanocarriers.^[94,95]^ As introduced in Section 3, several studies have demonstrated that lipid-based nanovectors might be good vehicles to improve the treatment of GBM. Lipid-based nanoparticles include liposomes, SLNs, and nanostructured lipid carriers (NLCs) **(Figure 4)**. The advantages that these systems present can be summarized in 1) the possibility of delivering hydrophobic and hydrophilic molecules,^[96]^2) the improvement of drug solubility,^[15]^ 3) a very low toxicity^[96]^ and safe biodegradation, 4) the increment of the half-life time of the drug in blood and also its action time,^[35]^ and 5) the control of the drug release, achieved by engineering the particles to respond to different stimuli.^[97]^ Moreover, the modification of the system surface can, on one side, avoid the recognition by the immune system and, on the other side, improve the specific targeting.^[98]^

### 4.1 Liposomes

Liposomes are spherical vesicles composed of a lipid bilayer, mostly phospholipids. This composition makes them biocompatible and biodegradable. Moreover, due to their amphipathic properties, they form vesicles in contact with aqueous solvents, improving the solubility and the stability of antitumoral drugs. As mentioned, both hydrophilic and lipophilic drugs can be encapsulated in the aqueous core or in the lipid membranes, respectively.^[48,94]^

Liposomes have been studied as possible drug delivery systems for the treatment of glioblastoma by numerous groups. For instance, Belhadj et al.^[99]^ developed a multifunctional targeted liposomal system, modifying the surface of liposomes with a cyclic peptide (arginine–glycine–aspartic acid, RGD) that preferentially binds to integrin α_*ν*_β_3_, overexpressed in BBTB and in glioma cells, and with *p*-hydroxybenzoic acid, to target the dopamine receptors of the BBB. This system carried the antitumoral drug doxorubicin. It was demonstrated in vivo with multiple doses administration that the nanosystem was better accumulated in the tumor area, showing a possible BBB and BBTB crossing, due to the dual targeting. Moreover, they showed an increase in the survival of the mice with respect to the free drug administration. In addition, they studied the safety of DOX-loaded liposomes, analyzing cardiotoxicity. While the administration of free DOX caused tissue degeneration, necrosis, and slight edema in healthy animals, mice treated with DOX-loaded liposomes did not present abnormal and inflammatory cell infiltration in heart or in other organs.

In another study, Jhaveri and collaborators^[100]^ developed PEGylated liposomes loaded with resveratrol, a potential drug against GBM, the use of which is hindered by its poor physical/chemical properties. To improve the targeting of the liposomes, Tf moieties were attached on their surface. The in vivo effects were evaluated in subcutaneous xenograft mouse models. It was observed that the size of the tumor was reduced in animals treated with six doses (one every 3 days) of the encapsulated drug with respect to the plain drug experimental group, although this difference was not statistically significant. On the other hand, they observed an increase in the survival rate of the animals from 16 to 28 days when comparing controls and Tf-liposomes, respectively.

In a recent study carried out by Zheng et al.,^[87]^ liposomes for the co-delivery of honokiol and disulfiram/copper complex have been exploited. The honokiol antitumoral effect is achieved by the inhibition of the PI3K/mTOR pathway. mTOR is a central pathway regulating the tumor microenvironment and its activation promotes GBM growth.^[101]^ The antitumoral effect of the disulfiram/copper complex has been explored in different clinical studies.^[102–104]^ To achieve dual targeting, liposomes were functionalized with a peptide that binds to the nicotinic acetylcholine receptors. This system was proved in orthotopic U87 and C6 glioma-bearing mice, and the formulations were administrated intravenously every 2 days five times. In C6 glioma-bearing mice, the survival time increased from 17 days (free drug) to 27 days (encapsulated drugs). Moreover, 30% of the mice survived until the end of the study, and when the possible toxicity of liposomes was studied in other organs by hematoxylin and eosin staining, no pathological changes were observed. In nude mice with U87 glioma, liposomes with the targeting moieties also exhibited an effective antitumor effect, with an increase in the survival rate of the mice, probably due to an improved targeting.

These kinds of systems are already being studied in clinical trials for the treatment of GBM,^[105,106]^ being the oldest studies based on the encapsulation of drugs such as daunorubicin^[107,108]^ or doxorubicin.^[109]^ Recent studies have focused not only on the encapsulation of therapeutic agents, but also on their specific targeting. For example, 2B3–101 is composed of PEGylated liposomes and doxorubicin with a targeting ligand for glutathione transporters.^[110]^ Phase I/II clinical trials have been completed in patients with solid tumors, metastatic brain cancer, or malignant recurrent gliomas (NCT01386580). Another example is the SGT-53 system. In this case, cationic liposomes have a p53 tumor suppressor plasmid that is targeted by a TfR ligand.^[111,112]^ The study showed minimal side effects in patients with advanced solid tumors. In addition, they observed the accumulation of the transgene in the tumors, demonstrating an effective targeting.

### 4.2 Solid Lipid Nanoparticles

SLNs represent a class of colloidal drug delivery system composed of physiological lipids in the solid phase at room and physiological temperature. SLNs are 50–1000 nm in diameter and present excellent stability, biocompatibility, easiness, and low cost of fabrication, besides low or no toxicity.^[96]^

In a study conducted by Kadari et al.,^[61]^ SLNs were used to encapsulate docetaxel. To improve the targeting, angiopep-2 was grafted on the surface of the nanomaterial. The in vivo study was conducted in C57BL/6 mice with injection of GL261 mouse glioma cells. They studied the biodistribution and the antiglioma efficacy of their system compared with free drug, using 10 mg kg^–1^ of docetaxel every 3 days for 3 weeks. They observed that the half-life time of the drug in blood was increased when encapsulated; moreover, the encapsulated docetaxel was better accumulated in the glioma, and the survival time of the mice increased from 24 days, in mice treated with the free drug, to 39 days, in mice treated with the drug-loaded SLNs presenting the targeting moiety. They analyzed the weight of the mice, as an indicator of systemic toxicity, and observed that mice treated with SLNs present a minimal loss of body weight.

Erel-Akbaba et al.^[113]^ designed SLNs for the delivery of interfering RNAs against EGFR, a receptor known to modulate tumor cell proliferation, viability, and differentiation, and against PD-L1, a transmembrane protein involved in immune checkpoints overexpressed in several tumors. To enhance targeting, CPPs were attached.^[114]^ This study was conducted in C57BL/6 mice bearing GL261 xenografts; before administrating the treatment, mice were irradiated, as it has been shown that radiation therapy alters the tumor for enhanced nanotherapeutic delivery.^[115,116]^ Thus, they demonstrated that mice that received radiation therapy in combination with multiple doses of their proposed drug delivery system showed a reduced glioblastoma growth and an increased survival. They confirmed by ex vivo histological examination that the expression of PD-L1 was reduced, whereas an increased CD8^+^ T-cell recruitment occurred, improving the immune response in the tumor.

A recent study performed by Wang et al.^[117]^ used a new anti-glioma oral prodrug called 13a-(S)-3-pivaloyloxyl-6,7-dimethoxyphenanthro(9,10-b)-indolizidine (CAT3), that presents a potent antitumoral effect against temozolomide-resistant gliomas in vivo.^[118–120]^ The drug was conjugated with a novel oleic acid formulation to increase its lipid solubility. This conjugated drug was encapsulated in SLNs and tested with a single-dose oral administration in healthy rats to study its pharmacokinetic. They measured the concentration of CAT3 and its metabolite PF403 in plasma. Rats that were administrated with CAT3-SLNs presented a lower concentration of CAT3 in plasma, which could mean a better transformation of the prodrug CAT3 into its active metabolite. When assessing the concentration of PF403, an increment in rats treated with CAT3-SLNs was found.

As it can be observed, SLNs are promising drug delivery systems, but they present various disadvantages such as a moderate drug loading capacity and a drug expulsion due to the process of crystallization under storage conditions.^[98,121,122]^

### 4.3 Nanostructured Lipid Carriers

These structures represent a second generation of lipid-based nanocarriers, that combine solid and liquid lipids at room temperature. In this way, the encapsulation capacity is improved, and the expulsion of the drug during the storage is avoided. In these formulations the most frequently used liquid lipids are glycerol trycolrylate, ethyl oleate, isopropyl myristate, and glycerol dioleate.^[123]^ The size of these structures is similar to those ones of SLNs, varying from 10 to 1000 nm in diameter.

The use of this system for the treatment of glioblastoma has gained attention in the last years. Song et al.^[124]^ developed NLCs to transport temozolomide. To improve targeting, they used the RGD peptide with high binding efficiency to endothelial cells of tumor vessels and glioblastoma cells. In in vivo studies, U87MG cells were subcutaneously injected in BALB/c nude mice. In the case of RGD–temozolomide–NLC multiple dose administration, the tumor size was reduced to more than 80%, three times higher with respect to the animal treated with plain drug.

Two years later, Zhang and collaborators^[125]^ demonstrated that temozolomide- and vincristine-loaded NLCs with dual targeting composed of the RGD peptide and lactoferricin were able to reduce the tumor size in BALB/c nude mice. They conducted intravenous injections of the nanosystem every 3 days for 21 days, obtaining an increment of the accumulation of the drugs into the brain and, on the other hand, a reduction of the concentration of the drugs in other tissues such as heart and kidney. The tumoral inhibition in the mice treated with this delivery system seems to not have systemic toxic effects, considering that the weight of the mice was not reduced.

In a recent study conducted by Basso et al.,^[126]^ atorvastatin and curcumin were encapsulated in NLCs. NLC surface was modified with folic acid and with cRGDfK for dual targeting, and with the peptide H_7_K(R_2_)_2_ to specifically target the acidic tumor microenvironment of GBM cells. The H_7_K(R_2_)_2_ peptide has a pH-responsive behavior due to the histidine residues (H_7_), that under acidic conditions present imidazole ring protonated.^[127]^ These ligands were conjugated with hyaluronic acid, and used for NLC decoration. The delivery system was tested in an orthotopic xenograft model with a single-dose administration and resulted in more specific targeting to the brain when the ligands are used, jointly to a reduction of the size of the tumor.

## 5 Lipid-Based Nanoparticles Combined with Magnetic Nanoparticles

As we have seen, lipid-based nanoparticles can be extremely useful as drug delivery systems. Different therapeutics agents can be encapsulated: from drugs such as tomozolomide, doxorubicin, docetaxel, or resveratrol to siRNAs; moreover, their surface can be easily modified to add targeting moieties, which improves the delivery of the drug to the nervous system. In addition, another advantage that lipid-based particles present is the possibility to use them as matrix for the complexation with other nanomaterials. In this section we will analyze recent studies where magnetic nanoparticles, and in particular superparamagnetic iron oxide nanoparticles (SPIONs), have been encapsulated within lipid-based nanocarriers.

Even though most of the studies concerning magnetic lipid nanovectors have been conducted in vitro, this field is of great scientific interest due to the advantages these systems present. On one side, we can take advantage of the lipid-based nanoparticle features, such as the high drug payload, the better targeting, and the improvement in terms of systemic toxicity of the treatment. On the other side, we could benefit from the magnetic nanoparticle characteristics:^[128]^ they can be used for imaging as contrast agents in magnetic resonance imaging (MRI),^[129]^ to perform physical targeting,^[130]^ and to induce magnetic hyperthermia^[131]^ applying an alternate magnetic field (AMF), and thus inducing a local temperature increment. This increased temperature can be beneficial for different purposes, such as the controlled drug release using temperature-sensitive nanomaterials. Moreover, depending on the temperature, the elimination of cancer cells, which are more sensitive to high temperatures than normal cells,^[132]^ can be done by hyperthermia or thermoablation. In hyperthermia, cells achieve a temperature of ≈44 ºC, which leads to perturbation of the intracellular microenvironment that affects the cellular functions and triggers apoptosis. In contrast, thermoablation reaches higher temperatures (>46 ºC) with consequent necrosis, coagulation, or carbonization of the tissues in a few minutes.^[133]^

In our group we proposed magnetic lipid nanoparticles for the treatment of glioblastoma. In 2019,^[134]^ we developed a lipidbased magnetic nanovector for the delivery of temozolomide. We showed that there was an improved release of the drug after the application of an alternating magnetic field; moreover, we demonstrated by an in vitro model of the BBB that these vectors could cross the BBB, besides confirming their capacity to use them in hyperthermia. A synergic combination of magnetic hyperthermia and of controlled release of the drug was demonstrated. In the same year, we added to the system an antibody against transferrin receptor as a targeting moiety, demonstrating in vitro, using tumor spheroids, that the targeting was improved and transcytosis from endothelial cells to the spheroids occurred. As a proof of concept, the nanoparticles were injected into a bovine post-mortem brain, and after the application of an AMF, the increment of temperature was monitored until it was stabilized at 42.5 °C.^[135]^

A similar system based on magnetic SLNs was used to encapsulate nutlin.^[136]^ In this case, an in vitro model of the BBB was developed combining in the same device the capability to recreate the blood flow and the possibility of studying the ability of the vectors to cross the BBB.

In a very recent study,^[60]^ a step further was conducted, with the functionalization of nutilin-3a-loaded magnetic NLCs with angiopep-2. Angiopep-2 could promote transcytosis through the BBB and could target the overexpressed receptors of glioma cells, improving the targeting. A microfluidic system was used to verify the efficacy of the platform and provide a demonstration that the vector was able to cross the in vitro model of the BBB, maintaining its targeting capacity toward glioma cells. Moreover, after the application of an AMF, it was observed that the temperature increment induced a permeabilization of the endo/lysosomal membranes, triggering the apoptosis pathway and killing cancer cells. In addition, an improvement in the drug efficacy was shown, demonstrating a synergic effect between hyperthermia and the drug.

Liposomes have been used to encapsulate SPIONs. The study conducted by Malinge and collaborators^[137]^ exploited SPIONsloaded liposomes doped with gadolinium-based positron emitters for positron emission tomography (PET) and MRI into a U87MG ectopic mice model. The targeting to solid tumor in mice was done using small neodymium magnetic discs in tumors and adding glucose moieties on the liposome surface. They could monitor and quantify the liposome uptake by both MRI and PET. Concerning MRI, they compared magnetic liposomes with an external magnet and magnetic liposomes with glucose moiety with or without the magnet. In all cases, they could qualitatively show accumulation in the tumor, demonstrating both the magnetic and the glucose targeting. For PET imaging, they administrated magnetic liposomes with gadolinium and with or without glucose moiety. To analyze the magnetic targeting, they placed a magnet in the tumor of the right leg, demonstrating that tumors in proximity of the magnet underwent a higher accumulation of nanoparticles with respect to those ones non-exposed to the magnets. The ex vivo analysis also confirmed the magnetic targeting: a 134% increment in the targeting efficacy in the magnetic liposomes was demonstrated when an external magnet was provided. When analyzing the magnetic liposomes with the glucose moiety, just a 32% of increase due to magnet targeting was observed. According to the authors, this could be possible due to the formulation of the system: when the magnetic liposomes with gadolinium and glucose are administrated, most of the nanovectors stay in the injection site. The viscosity of the formulation is higher, which makes the injection more difficult and the loss of a large part of the radiotracer.

Babincová et al.^[138]^ used rats with C6 glioma xenografts to test the efficacy of magnetoliposomes loaded with doxorubicin. After 45 min since particle injection, they applied a magnetic field. This process was repeated twice a week for 28 days and they observed that the volume of glioma was reduced from 3.7 cm^3^ (control rats) to 0.2 cm^3^, approximately.

In addition to these in vivo studies, interesting proposals are being developed that have obtained so far promising results in vitro. An example is the study conducted by Shi et al.,^[139]^ that developed a thermosensitive liposomal system composed of liposomes loaded with magnetic nanoparticles and doxorubicin. To enhance the targeting, they used two moieties: CPP specific for GBM (P1NS) and an anti-GBM antibody (TN-C). Results showed that the system was able to cross an in vitro model of BBB, displayed a thermoresponse, had a glioblastoma-specific cellular uptake, and had a controllable drug release profile. Moreover, they observed that these magnetoliposomes caused suppression of cell proliferation in U87 cultures, without causing any significant impact in healthy cell function.

Another promising study conducted by Anilkumar et al.^[140]^ investigated cationic liposomes containing citric acid-coated iron oxide magnetic nanoparticles for magnetic hyperthermia and photothermia, induced by AMF and near-infrared laser, respectively. In this study, the authors first demonstrated a synergic hyperthermia due to both AMF and laser stimulation; later, they studied the effect of liposomes in U87MG cells. It is described that cationic liposomes can accumulate due to their positive charge,^[141]^ thus entering in cells via charge-mediated endocytosis. Once accumulated in cells, researchers analyzed the viability of the cultures after AMF or near-infrared laser treatment. They demonstrated dosedependent toxicity with no differences in cell death when using AMF or near-infrared laser separately. However, they could show a significant reduction in cell viability (≈30%) when using both AMF and laser together, demonstrating the advantages of using combined magnetic and photothermal hyperthermia.

These studies, shown in **Table 1**, led us to the conclusion that lipid-based nanosystems, together with other nanomaterial such as magnetic nanoparticles, can indeed be a promising tool in the treatment of glioblastoma.

## 6 Conclusions

The treatment of GBM is one of the most challenging research topics in cancer. As we have seen in this Review, different approaches are being studied using lipid-based nanosystems. They present various advantages with respect to other nanomaterials, including biocompatibility, the possibility of modifying their surface to enhance the cell targeting, and their intrinsic ability to cross the BBB. Nevertheless, the majority of the research has been done in vitro or in animal models, and still a few strategies have involved clinical trials. To overcome this issue, it is necessary to improve the reproducibility of the nanosystems in contact with a biological environment. We need to deeply control and understand 1) the physical/chemical properties of the nanoparticles, 2) how they interfere with the biological milieu (blood, for instance), and 3) the interaction with the biosystem. In recent years, a great effort has been made to know the physical/chemical characteristics of these systems. However, more research should be performed in the targeting strategies of the nanoparticles, especially if the intravenous administration route is used: it is known that less than 1% of the administrated nanocarriers reach solid tumors.^[142]^ So, in the near future, we need to focus on understanding the fundamental interactions of nanoparticles with organs and tissues where they accumulate or are eliminated.

Another fact that can reply to the lack of clinical translation may be the preclinical model itself. Analyzing the different in vivo models, we observe that the chemically-induced tumors are quite different from human GBM, and in fact, although different components have been shown to cause glioma tumors in animals, there is not a single chemical compound related to the development of human GBM. It is important to carefully consider the histopathological characteristics as well as the molecular signals of these types of tumors, to not overestimate the therapeutic efficacy of the treatment. Moreover, the use of xenograft transplants also presents some disadvantages: the major limitation of these models is that unlike in patients where the transformation of a single cell triggers the tumor, in this preclinical model a huge number of cells have to be implanted. The need to first grow these cells in vitro may lead to genotypic and phenotypic deviation from the original cells, which decreases the reproducibility of the results. In addition, the genetically modified mice do not reflect the inter- and intra-tumoral heterogeneity that is observed in human GBM. Thus, it is important that in the future we will be able to reproduce human GBM models more precisely and closely. If we are not able to generate better predictive models, different existing models could be used simultaneously to ensure a better approximation to reality. Despite the disadvantages of these models, there are already cases where liposomal formulations have been used for the treatment of GBM in clinical applications;^[4]^ however, studies for SLNs or NLCs are scarce or missing at all. We believe this could be due to the fact that these systems are newer and have not had enough time to be widely tested yet. Improving the preclinical models and making extensive investigations on more realistic situations will lead to better understanding the interactions of SLNs and NLCs with living matter, thus increasing the hopes that patients might be able to benefit soon from these new drug delivery systems, improving the quality of their life as well as their survival rates.

## Figures and Tables

**Table 1:** Summary of different studies conducted with lipid-based nanocarriers combined with magnetic nanoparticles.

Nanomaterial	Targeting moiety	Drug	In vitro model	In vivo model	Main finding	Ref.
SLNs	–	TMZ	BBB model	–	Improvement of the drug release after the application of an AMF Crossing of the system through the BBB model Hyperthermia	[134]
SLNs	Transferrin	–	Tumor spheroids	Bovine post-mortem brain	Improvement of the targeting and achievement of transcytosis Increment of the temperature inside brain tissue after AMF application	[135]
SLNs	Magnet	Nutlin	BBB model	–	Demonstration of the ability of the system to cross the BBB model	[136]
NLCs	Angiopep-2	Nutlin	BBB model	–	Glioma cell targeting and transcytosis through the BBB model Cell death by hyperthermia after the application of an AMF Improvement of the drug release	[60]
Gd-liposomes	Glucose Magnet	–	–	U87MG ectopic model	Improvement of the tumor targeting by the magnetic implants and the glucose moiety	[137]
Liposomes	–	DOX	–	C6 glioma-bearing rats	Reduction of the tumor size after the application of the AMF	[138]
Liposomes	CPP GBM-specific antibody	DOX	BBB model U87MG cell line	–	Crossing of the BBB model and targeting of glioma cells Controlled drug release by temperature Suppression of U87MG cell growth	[139]
Cationic liposomes	Positive charge	–	U87MG cell line	–	Improvement of cell death after the application of AMF together with near-infrared laser	[140]
